# Supplementary material for: Development of a Rapid and Cost-Effective Multiplex PCR Assay for the Simultaneous Identification of Three Commercially Important Sea Squirt Species (Halocynthia spp.)
Source: Foods. 2025 Aug 27;14(17):3003. doi: 10.3390/foods14173003 (PMC12427941; doi:10.3390/foods14173003)
Supplement: Supplementary file 1 [file foods-14-03003-s001.zip › foods-3797183-supplementary.pdf]

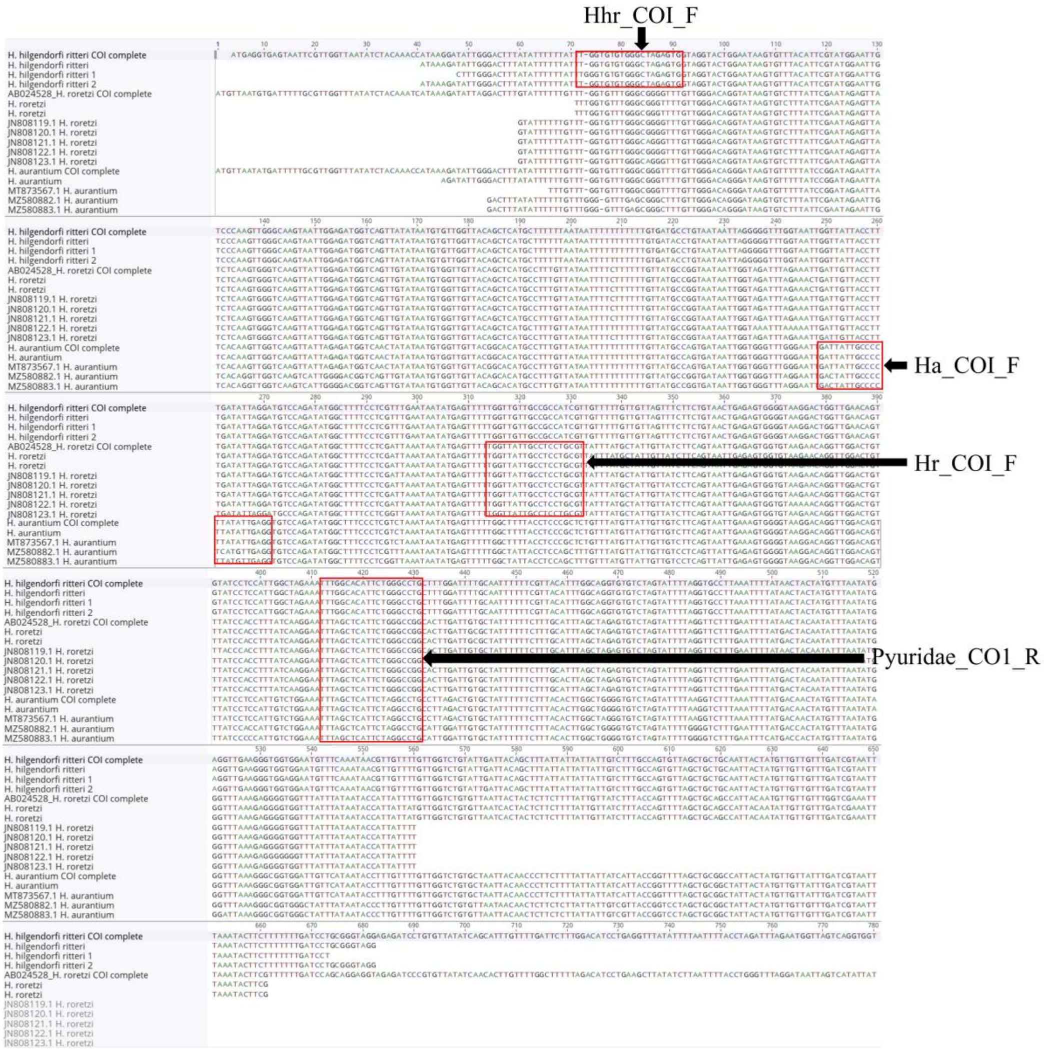

Figure S1. Species-specific primer locations using COI partial for three species of sea squirts. The "COI complete" sequences shown in the figure were extracted from each species mitochondrial genome (AB024528, MT811760, and NC_053533). Sequences with only the species name are extracted using universal primers (LCO1490 and HCO3198). Hhr_COI_F, Ha_COI_F, and Hr_COI_F are species-specific primer sequence names provided in Table 1.

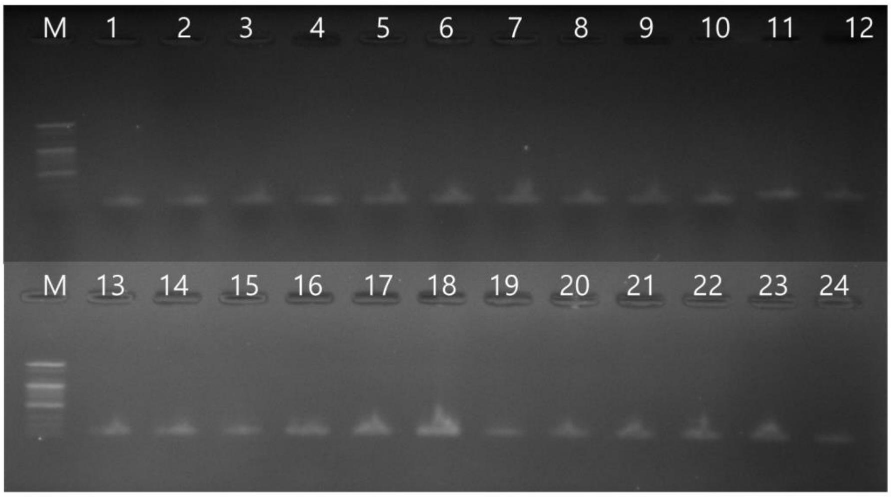

Halocynthia roretzi (118 bp) individuals

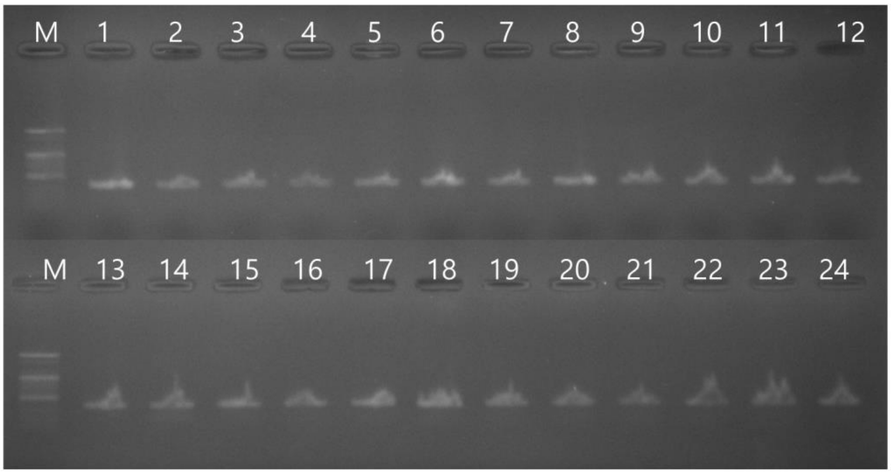

Halocynthia hilgendorfi ritteri (118 bp) 24 individuals

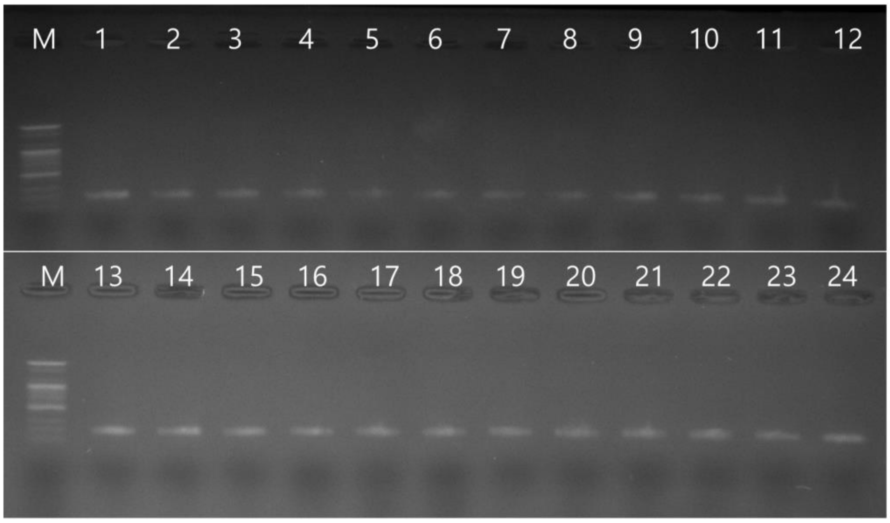

Halocynthia aurantium (186 bp) 24 individuals

Figure S2. Twenty-four individual bands for three species of sea squirts. Each band is a PCR amplification product using a multiplex PCR assay.

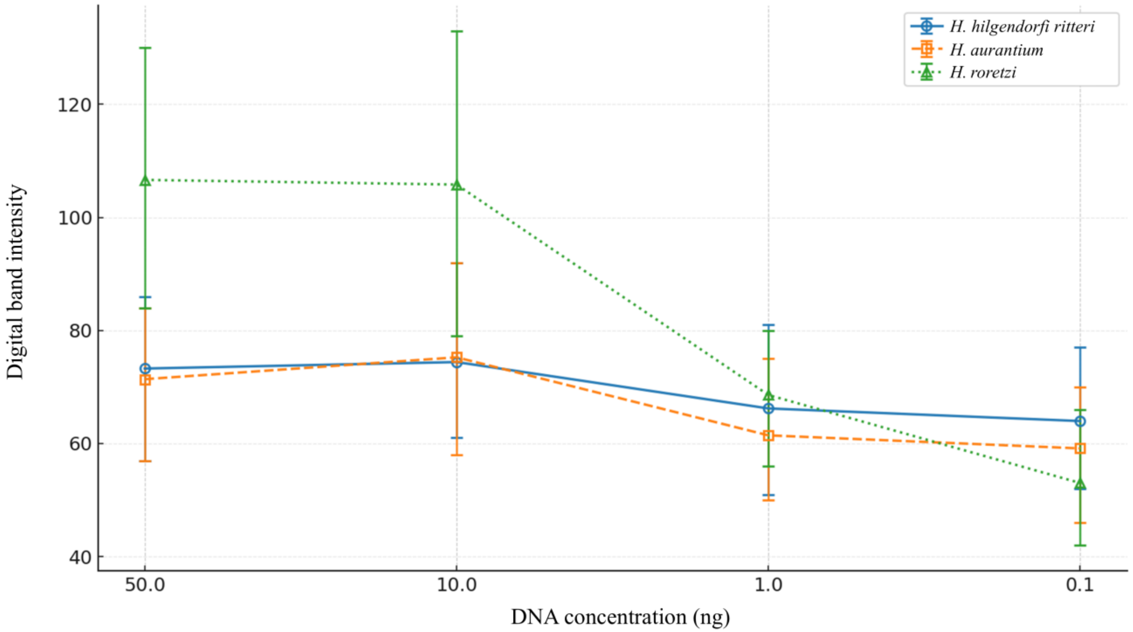

Figure S3. Digital quantification values of the bands for three species of sea squirts. Each line color represents the digital quantification value for that species. Error bars indicate the minimum and maximum values, and the center line indicates the mean.
